# A grounded theory of the process of social transition in a family context from the perspective of gender diverse young people and their parents

**DOI:** 10.1111/camh.70078

**Published:** 2026-02-27

**Authors:** Sophie Razzel, Simon Levinson, Trish Joscelyne

**Affiliations:** ^1^ Salomons Institute for Applied Psychology Tunbridge Wells UK; ^2^ Great Ormond Street Hospital London UK

**Keywords:** Social transition, transgender, families, adaptation, resilience, gender minority stress

## Abstract

**Background:**

Some gender diverse young people (GDYP) socially transition—a process of changing from living as the gender that they were assigned at birth to another gender, through social means. Whilst the role of the whole family in the social transitions of GDYP has been indicated, there is a lack of applicable theory and research on the processes of social transition within UK families. The aim of the current study was to address this gap by building a grounded theory of the social transition of young people within the family context.

**Methods:**

We conducted one‐to‐one, semi‐structured interviews with nine participants from four UK families (four GDYP and five parents). All participants were recruited through the UK Gender Identity Development Service (GIDS). Data were analysed using grounded theory methodology.

**Results:**

The developed theory suggests that the social transition of young people within the family context is a young person‐led family resilience process, through which families work towards the young person experiencing greater acceptance and belonging. The process consisted of several adaptive subprocesses and was influenced by contextual factors. In managing the challenges of social transition and particularly non‐affirming responses, families increased in resourcefulness and developed the understanding and tolerance of their communities.

**Conclusion:**

Further research is needed, particularly in families of different racial backgrounds, and to assess the applicability of the model to families without an affirming parent. Implications are considered within the context of recent changes in UK clinical service provision and developments in educational guidance.

**Key Practitioner Message**

**What is currently known?**

- Many gender diverse young people socially transition. The previous evidence base indicates that this may have important implications for well‐being and is a family‐level process. There was a previous lack of relevant theory and research to explain the ways in which well‐being, social transition and the family are connected and the processes of social transition within UK families

**What has been shown?**

- Social transition within the UK family context appears to be a family resilience process, through which families work towards their young person experiencing greater acceptance and belonging. Exposure to gender minority stressors and the associated challenges of this exposure requires families to adapt through a process of developing family resilience

**What is the significance of this for clinical practice?**

- Clinical and educational practitioners should attend to the impacts of gender minority stressors when working with gender diverse young people and their families. Assessment and intervention should be routinely offered at the family level. Further research is needed to understand how the processes of social transition occur in families with other characteristics, and to attend to the ways in which changes in services and policies will likely impact social transition and associated well‐being

## Introduction

There has been a significant increase in gender‐diverse children and young people (GDYP) presenting to UK mental health and National Health Service (NHS) gender identity services in recent years (Cass Review, 2024). Whilst the application of a diagnostic framework to this population can be seen as pathologising and contributing to victimising narratives, GDYP do face a significantly increased risk of several negative health outcomes, including increased anxiety, depression and suicidality (American Psychiatric Association, 2022; Horton, 2020; Olson‐Kennedy et al., 2016).

### A note on language

*GDYP* is the term used in this study to refer to children and young people (CYP) whose gender identity, role and/or expression differ from that which is culturally expected for them. This may include transgender and/or non‐binary young people, as well as those of other gender identities (Mermaids, n.d.; American Psychiatric Association, 2015). The authors do, however, wish to highlight the significant complexities in selecting a term to describe this population, and the importance of self‐determination in language choice. The term *cisgender* is used to describe individuals whose identified gender corresponds with that which they were assigned at birth (American Psychiatric Association, 2015).

### UK context

The Cass Review was a recent independent review of gender services for CYP commissioned by NHS England and NHS Improvement (Cass Review, n.d.). The interim report resulted in the closure of the Gender Identity Development Service (GIDS) and service restructuring (Stewart & Palmer, 2024). This, along with the published final report, has received considerable public attention within a pre‐existing context of controversy and public debate around the identities of GDYP, which formed part of the context during the time this research was carried out. Additionally, and partly informed by the interim Cass Report, the Department for Education recently published draft non‐statutory guidance for schools and colleges on supporting ‘Gender Questioning’ Children (Department for Education, 2023; Department for Education, Badenoch, & Keegan, 2023). Whilst this was published following our data collection, it has relevance to the study implications as it appears to place parameters around social transitioning in schools, and school‐based experiences were salient in the social transitions of the young people in the present study.

### Social transition and the family

In the United Kingdom and internationally, many GDYP presenting at services have undergone social transition. Social transition may include changing name, pronouns, use of facilities and clothing, but does not include medical transition, although these processes may occur alongside one another (Hall et al., 2024; Cass Review, 2024). It has been suggested that social transition can be associated with a reduction in mental health distress experienced by GDYP, and supporting social transition has been understood as aligned with gender‐affirming care (Ehrensaft, Giammattei, Storck, Tishelman, & Keo‐Meier, 2018; Hall et al., 2024; Kuvalanka, Weiner, & Mahan, 2014). The wider evidence base exploring associations between social transition and well‐being outcomes does, however, appear to be somewhat mixed (Durwood, McLaughlin, & Olson, 2017; Morandini et al., 2023).

Previous research has demonstrated that family responses significantly impact the ability of GDYP to socially transition (Schimmel‐Bristow et al., 2018), and family acceptance has been demonstrated to play an important role in GDYPs' psychosocial adjustment (Pariseau et al., 2019). Further highlighting this centrality of family, it has been suggested that social transition should be framed as a ‘family‐level’ event (Bull & D'Arrigo‐Patrick, 2018). Understanding the family experience of social transition is therefore important.

Previous social transition research has largely been conducted in the United States and Canada, with studies highlighting meaning‐making processes and young‐person led decision‐making (e.g. Bull & D'Arrigo‐Patrick, 2018; Olson et al., 2019). Given the current UK context, studies from elsewhere are likely to have only limited applicability, and most previous research has not included the voices of GDYP—a considerable limitation.

Despite the indicated possible role of social transition in the mental health of GDYP and the centrality of the family, there is also a lack of relevant theory to explain the processes by which these are connected. Existing systemic theories may offer some insights. Family resilience theory conceptualises *family resilience* as the family's capacity to positively respond to challenges through adaptation processes (Walsh, 2016). Catalpa and McGuire (2018) described how, in perpetuating the belief that cisgender identities are the norm, cisnormativity ‘disallows’ the existence of gender diverse people in families. This belief perhaps indicates that a young person's social transition could challenge a family, requiring adaptation that can be understood through family resilience theory.

GDYP are also likely to be exposed to experiences which can be understood as gender minority stressors, including peer victimisation, which is associated with increased mental health difficulties, and reduced school belonging (Hatchel, Valido, De Pedro, Huang, & Espelage, 2019; Hendricks & Testa, 2012; Hunter, Butler, & Cooper, 2021; Meyer, 2003). It seems likely that gender minority stress would also challenge family resilience. Notably, existing models of family resilience have not been grounded in research with GDYP, and there is a risk of further pathologising GDYP when making broader inferences (Horton, 2020; Toomey, 2021).

To address the above gaps in research and theory, we aimed to build a grounded theory of the process of social transition of young people within the UK family context. Four primary questions guided this:

1. How is social transition in young people understood by, and negotiated with, family?
2. What do young people and parents/carers view as the changes that occurred within the family as a whole as the young person socially transitioned, and how do they understand those now?
3. What is seen as helpful or hindering to the social transition of a young person within the family context?
4. What influence are racial identities, culture, class and community seen as having on how the family understands and negotiates the social transition of their young person?

## Methods

### Design

A qualitative, grounded theory design and the methodology of Corbin and Strauss (2015) were chosen, as this inductive approach allowed the voices of the GDYP to be centred in the developing theory and is accommodating of the researchers' critical realist positions. The emphasis of critical realism on giving voice to those often marginalised was also considered particularly appropriate given the minoritised identities of GDYP (Bhaskar, 2008; Hunter et al., 2021; Looker, Vickers, & Kington, 2021).

### Ethics

HRA/Health and Care Research Wales approval was granted (REC reference: 21/LO/0854). Informed consent was obtained for all participants aged 16 and over, and assent was obtained, with parental consent, for participants under 16. The ethical guidelines of Vincent (2018) for research with transgender people were also considered throughout, including careful consideration of language, maintaining transparency and ensuring researcher's knowledge of trans history and neglected discourses. Such nuance is important due to the historical exposure of transgender populations to some flawed research practices (Vincent, 2018).

### Participants

To allow access to this very specific population, all participants were recruited through the (now former) GIDS. In order to meet the study eligibility requirements, young people had to be aged between 11 and 18 years at the time of participation. Nine participants from four families participated: four GDYP, aged 12–17 (Mean = 15.5 years) and five parents, aged 36–58 years (Mean = 45.2 years). One parent from each family participated, except for Family 1, in which two parents participated. See Table 1 for detailed participant characteristics with pseudonyms. All participants were invited to choose their own pseudonym should they wish to; pseudonyms were therefore either self‐selected by the participant or otherwise chosen randomly by researcher SR.

**Table 1:** Self‐identified participant characteristics (in interview order)

(Family number) and pseudonym	Relationship to GDYP	Gender identity	Pronouns	Age (at time of interview)	Ethnicity
(1) Zoe	Mum	Cisgender female	She/her	36	White British
(1) Dan	Biological father	Straight male	He/him/his	39	White British
(1) Ariana	Young person	A girl	She/her	12	White British
(2) Ruby	Young person	A girl, and I will be a woman	She/her	15	White British
(2) Lori	Mother	There's no male and female, I'm just [Lori] and that's it.	She/her	49	White British and human!
(3) Guitar	Young person	Very masculine. Always hated feminine things, things to do with girls	He/him	17	White
(3) Sarah	[Guitar's] mum	Female	She/her	44	White British
(4) Zac	Young person	Trans male/Trans man/Trans Guy	He/him	18	White British
(4) Elly	Mother	Woman	She/her	58	White British

### Service user consultation

Members of a GIDS stakeholder group, including both GDYP and parents/carers of GDYP, reviewed the suitability of the research materials. Feedback was carefully considered, with changes made accordingly.

### Procedure

Following a request from the researchers, GIDS clinicians identified families considered suitable from their caseloads and approached participants directly, providing information and requesting they contact the researcher by email if interested. Purposive sampling was used for the initial round of recruitment according to the inclusion criteria. Following initial recruitment, data collection and analysis, theoretical sampling was used to recruit further participants with certain characteristics, allowing the categories to be more fully developed.

All interviews took place individually via video call on a one‐to‐one basis with the lead researcher. The interviews were audio‐recorded and guided by interview schedules with some deviations to support theory development. GDYP and their parents were asked similar questions, but with slightly amended wording to capture their differing roles. Recordings were transcribed verbatim by SR and analysed, beginning with open‐coding after the first interview. Constant comparison, axial coding with diagrams, selective coding and diagramming were all applied. Given scale and time constraints, data collection could not be pursued open‐endedly and theoretical sufficiency was therefore aimed for, with the intention of achieving conceptual depth (Dey, 1999; Nelson, 2017).

### Reflexivity, positionality and quality assurance

Best practice guidelines for conducting qualitative research were used (Yardley, 2000). To support reflection on assumptions, biases and emotional processes, SR completed an audio‐recorded bracketing interview, kept a research diary and engaged in supervision throughout. These processes included ongoing reflection on how SR's own identification as Queer and non‐binary and without children may have influenced their engagement with the research, as well as their identified inclination to attend more to the GDYPs' views than those of parents. Theoretical memos were also kept, documenting the process of theory development, whilst SR primarily completed the analysis, this was reviewed by all authors. All participants were later invited to review and comment on a report of the research, and three responded, expressing agreement with the model.

## Results

### Model overview

The theory explaining the process of social transition within the family context was described by the overarching category: *Working towards a place of acceptance and belonging*. This was a young person‐led family resilience process, through which families worked through multiple adaptive subprocesses, beginning within the family context before changes outside of the family. Table 2 provides an overview of the organisation of categories. Figure 1 shows the relationship between the categories and the final theoretical model.

**Table 2:** Organisation of categories and subcategories

Overarching category: Working towards a place of acceptance and belonging	
Main category	Subcategories
1. Main category: Recognising and accepting this young person	(1.1) Gender identity is already there
	(1.2) Relational recognition
	(1.3) Developing understanding
	(1.4) Managing disclosure
	(1.5) The importance of transition
2. Main category: Negotiating the changes inside and outside the family	(2.1) Anticipating reactions
	(2.2) Making requests
	(2.3) Non‐affirmation
	(2.4) Affirmation
3. Main category: Negotiating relationships inside and outside the family	(3.1) Negotiating relational difference
	(3.2) Distancing
	(3.3) Preserving and increasing connections
4. Main category: Adaptation and resilience	(4.1) The emotional challenge
	(4.2) Coping: strengths, skills and strategies
	(4.3) Fighting strategies
	(4.4) Resourcing: accessing the necessary support (4.5) Developing self (4.6) Developing community
5. Main (Contextual) category: Social contexts and shaping characteristics	(No subcategories)

**Figure 1:**
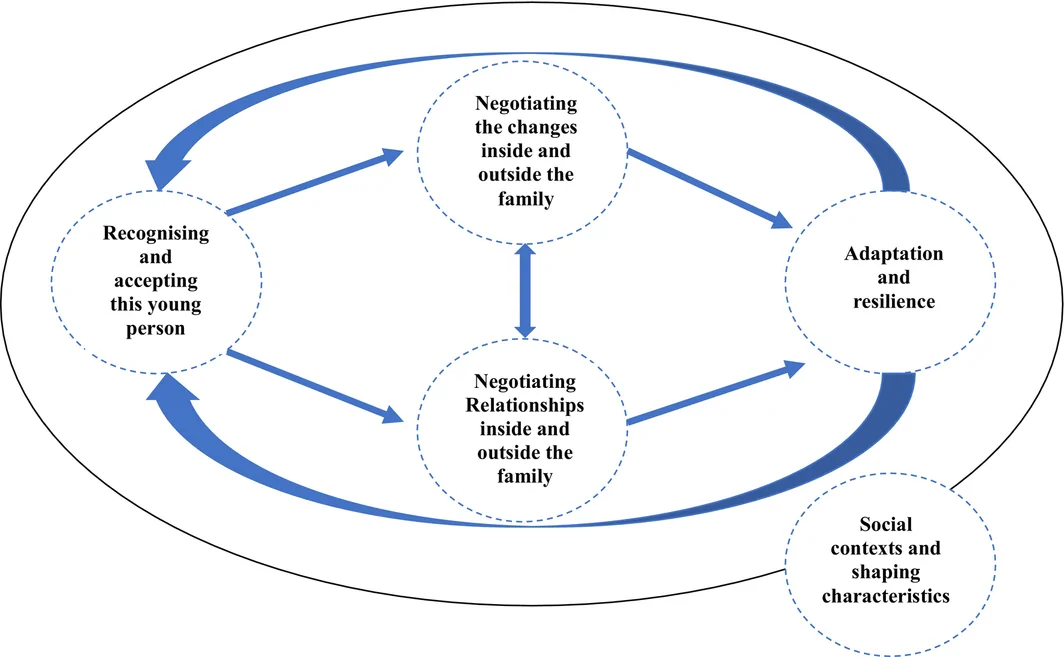
A model of gender social transition of young people within the UK family context; interaction of the main categories

### Main category 1: Recognising and accepting this young person

The social transition process began with developing recognition and acceptance of the young person's gender identity.

#### Subcategory 1.1: Gender identity is already there

All the families understood the young person's gender identity as having long been present, including before recognition: ‘…before I knew what trans was, I think, that was when I was trying to present myself to be more masculine’ (Guitar, young person, age 17).

#### Subcategory 1.2: Relational recognition

Recognition appeared to develop relationally. For example, Ariana's (young person, age 12) social transition began with dressing up with her cousin: ‘…I used to dress up with her’. Family members' processes of recognition were also interrelated. For example, Zac (young person, age 18) described: ‘…the more I was showing like questioning of it [gender identity] and the more uncertain I was, I feel like that reflected in them [mum] as well’.

#### Subcategory 1.3: Developing understanding

As different family members developed understanding, they recognised and accepted the young person's gender identity at different times. For example, regarding his initial recognition of his daughter Ariana's gender identity, Dan described how it took him ‘a few months to sort of process everything’.

#### Subcategory 1.4: Managing disclosure

Whilst the young people's early gravitation towards preferred items, such as toys and clothing typically associated with a different gender, had usually been accepted by the family, the young person also later expressed their wish to make certain changes. Young people appeared to first communicate this to the family member they deemed as most likely to accept their gender identity. In communicating this wish for change, the young person also disclosed their identity. Accounts highlighted the importance of the young person experiencing acceptance from close others regarding their authentic gender identity and expression: ‘I wanted to be sure that we [me and mum] were in agreement, and we were’ (Guitar, young person, age 17).

#### Subcategory 1.5: The importance of transition

Families came to recognise the importance of supporting social transition, and of the process being child‐led. This included Guitar's (young person, age 17) suggested advice for parents:‘… just let them [GDYP] lead’. Social transition was understood as allowing the young people to fully express themselves: ‘…to allow them to kind of flourish and express who they are, without any restriction’ (Dan, Ariana's father), as well as enabling them to experience belonging: ‘it's sort of him finding his place in the world’ (Elly, Zac's mother).

### Main category 2: Negotiating the changes inside and outside the family

Beginning within the family and then implementing changes within other contexts, the families negotiated changes of social transition and associated responses.

#### Subcategory 2.1: Anticipating reactions

Participants usually anticipated others' reactions. For Guitar (young person, age 17), this began with his parents: ‘I think I did have… maybe like a fear that my dad or, and my mum, would sort of just think that, you know, it's a phase, I'm not, I'm not gonna let you do it…’. Contrastingly, anticipation of accepting reactions supported the process for Zac (young person, age 18): ‘I felt like extremely supported and extremely like, I almost could predict how they [mums] would react so it just made it a lot easier’.

#### Subcategory 2.2: Making requests

Families began working towards acceptance and belonging in other contexts by requesting gender‐affirming changes. Families moderated the introduction of changes in efforts to increase the likelihood of acceptance. For example, regarding changes to the school uniform she wore, Ruby (young person, age 15) described: ‘[Mum said] “we won't do the skirt just now because I think it's best if you ask the school or I ask the school’”. Guitar's family also moderated changes as others' reactions to these were anticipated; Guitar's mum, Sarah recalled: ‘… I was aware of, you know, how this (Guitar's gender‐affirming changes) might be perceived by other people’.

#### Subcategory 2.3: Non‐affirmation

Non‐affirming reactions of others were common across many contexts: ‘[My grandparents] dead name me, misgender me …’ (Zac, young person, age 18). Ruby (young person, age 15) recalled her dad's response to her gender identity and expression: ‘…he was quite, like, mentally abusive to me. Like, he'd be like “No, like stop acting so gay’”. To manage this, Ruby tried to hide her gender expression until, following her parents' separation, she was able to express this more freely. Additionally, Ruby's mother Lori's own increased freedom following the separation informed this. Lori described: ‘… all of a sudden, I was free to be who I was without any restrictions. Why should I then enforce something on my child?’

Ruby's social transition seemed to be defined by experiences of non‐acceptance; she recounted many experiences of peers ‘being transphobic’ towards her at school, describing: ‘…transitioning in general you're sort of like being everyone's target’. These reactions appeared to undermine young people's belonging. For example, regarding peers, Ariana (young person, age 12) experienced: ‘…they didn't want to be in the same toilet as me’, and Sarah (Guitar's mum) described how Guitar's ‘…best friend rejected him’.

Difficult experiences with professionals seemed common. Recalling an experience in school, Ariana (young person, aged 12) described: ‘…I got so nervous about it, getting put into girls and boys. And I got put in the boys and I got so sad’. Ruby (young person, age 15) also had difficult experiences with professionals; seemingly responding with concern to her social transition, staff at her school made a referral to social services: ‘… social services got involved and said that mum was brainwashing me into thinking that I was a girl…it felt like a big interrogation’.

#### Subcategory 2.4: Affirmation

Several parents cited prioritisation of their young person's happiness as related to their accepting response: ‘I just want a happy kid. I don't care what gender she is’ (Zoe, Ariana's mum). Beyond immediate family units, experiences of affirmation were recalled, but less commonly than the non‐affirming responses. Ruby's (young person, age 15) reflection on rarer affirming experiences highlighted the contrasting power of affirmation. She became tearful when recalling receiving a gift during the earlier stages of her gender expression that had felt particularly gender‐affirming: ‘…I love her for this…. she got me these gorgeous blue pumps. I've still got them upstairs’.

### Main category 3: Negotiating relationships inside and outside the family

As the families negotiated the changes of social transition, relationships were negotiated and redefined.

#### Subcategory 3.1: Negotiating relational difference

Some parents appeared to take on a ‘buffering’ role between young people and other family members, such as Elly (Zac's mother), who described: ‘I've had to um, talk to his dad. Previously when he was young about, ‘please don't put him in really girly dresses’. Different family members’ needs were also negotiated. For example, Sarah (Guitar's mum) described: *‘bearing in mind that I'm a working mum, I have another child… I think sometimes my second child did miss out a little bit’*.

#### Subcategory 3.2: Distancing

Social transitions involved changes in the ways the immediate family unit interacted with extended family and other systems. Elly (Zac's mother) taking on a ‘buffering role’ and advocating for Zac appeared related to an increase in distance in her relationship with Zac's dad. She described: ‘…we (Elly and Zac's dad) don't talk anymore… probably because I've been the bad guy’. In some families, explicit estrangement was considered or used as a means of protecting the young people from non‐accepting responses. This occurred in Ariana's family: ‘…We don't speak to my dad's side of the family because they didn't really like me being a girl’ (Ariana, young person, age 12). Ruby's mum, Lori, described how she had been prepared for estrangement: ‘I was quite happy to cut off every single member of my family if it meant… [Lori begins to cry] if it meant that Ruby could be who she was supposed to be.’

#### Subcategory 3.3: Preserving and increasing connections

All the families experienced increased closeness within some relationships. Zoe (Ariana's mum) reflected: ‘…we're probably closer to be fair than anything because of, I don't know, the fact she is trans that, you know, she has gone through so much and she knows we've got her back’. Maintaining relational closeness was also an important strategy, as exemplified when Guitar (young person, age 17) considered advice he would give to parents/carers of other gender diverse young people: ‘…stay close it will really benefit you and the young person’. Whilst some non‐accepting responses led to relational distancing, in other instances, compromises were made to preserve relationships. For Ruby (young person, age 15), this involved negotiating with her brother when he did not accept calling her Ruby. Ruby recalled: ‘…So we came up with [alternative name]’.

### Main category 4: Adaptation and resilience

As they adapted to challenges, families developed capabilities and resilience, which equipped them in their continued work through other aspects of social transition.

#### Subcategory 4.1: The emotional challenge

Zac (young person, age 18) described the emotional family journey: ‘…like immediate family impacted us both, like us all. Like, mentally’; ‘it was that journey we all had to go on’. Participants also experienced mental health impacts related to the many examples of others' challenging responses. Guitar's mum, Sarah, described: ‘where he [Guitar] was kind of having these social responses from people, trying to navigate these pronouns, trying, you know, living this double life not being the authentic him… He tried to hang himself twice’. Regarding social services' involvement, Lori (Ruby's mother) described the: ‘… damage that it had done, to the family unit. And to my mental health as well’.

#### Subcategory 4.2: Coping: Strengths, skills and strategies

Participants used a range of strategies in managing difficulties. Zoe (Ariana's mum) tried to *‘shield’* and keep Ariana *‘out of the loop’* regarding some difficulties with school, and Ariana's father, Dan's account indicated that open communication within the family was also sometimes an important strategy:‘…if there's an instance at school, we talk about it’.

Cognitive strategies were also used. In managing difficult gender‐related experiences with peers, Ariana (young person, age 12) described: ‘I've just got to… let go of it and think “well, they're stupid” and… you know. “Just forget about them’”. This was perhaps a strategy learnt from mum, Zoe, who seemed to support Ariana to maintain a positive outlook, by advising: ‘…it's one person, just ignore them…’. Participants also drew upon past experiences and existing strengths. This included attributes of the young people both supporting and developing through social transition. For example, Ruby (young person, age 15) reflected: ‘I just one day I built up the courage, I was like “Mum I wanna be a girl’”.

#### Subcategory 4.3: Fighting strategies

Several families experienced a sense of having to ‘fight’ for acceptance of the social transition. Ariana's (young person, age 12) family experienced several requests denied by school staff: ‘…when she went to school, you just felt like… “God what el[se]? What next’” (Zoe, Ariana's mum). Acceptance of Ruby's social transition within secondary school also seemed to have required a significant ‘fight’. Lori (Ruby's mother) described: ‘… she was still forced to use male toilets and male changing rooms’. Lori seemed to use a strategy of ‘picking your battles’, recounting how she advised Ruby to compromise: ‘You're just gonna have to get on with it’.

#### Subcategory 4.4: Resourcing: Accessing the necessary support

Participants required and worked to source appropriate support. Sarah (Guitar's mum) described support as *‘key’* for parents, and: ‘the most critical thing to have, I think, personally’. The value of support from close friends and other family members was also described by Zoe (Ariana's mum): ‘…having family support is massive…’.

Accounts highlighted the benefits of support from a range of services and professionals, as well as unmet support needs. Schools appeared to hold important potential in supporting the social transitions. For example, regarding her primary school headteacher, Ruby (young person, age 15) reflected: ‘… she was really, really nice and put unisex toilets in, so everyone could go together’. Similarly, Ariana (young person, age 12) described her secondary school as ‘all for equality’, indicating the potential for whole school cultures to be places of acceptance and belonging for GDYP. Despite these positive experiences, significant difficulties and apparent school‐based barriers to social transition were salient across accounts. Additionally, Sarah (Guitar's mum) described significant difficulties in accessing child and adolescent mental health service support for Guitar, including when he was experiencing suicidality: ‘… [the psychologist] basically felt that I was giving Guitar adequate enough support at home. So they wouldn't see him’.

#### Subcategory 4.5: Developing self

Participants reflected on the ways their social transition experiences had shaped them, ascribing positive new meanings. For example, Ariana (young person, age 12) reflected on how coping with others' reactions shaped her: ‘…it's made me who I am’. Similarly, Zac (young person, age 18) described: ‘…my identity is something I hold close to my heart because I had to go a long way to get to where I am…’.

#### Subcategory 4.6: Developing community

Families also supported the development of their communities, including by educating others. Lori (Ruby's mother) described conversations she has with work colleagues: ‘I always then use that opportunity to educate them (regarding gender diversity)’. Zoe (Ariana's mum) reflected on Ariana's friendship circle: ‘…there's like, doesn't seem to be any prejudice around it. But I think, I don't know whether we've like sort of paved the way a little bit’. Similarly, regarding the challenges negotiated with Ariana's school, Dan (Ariana's dad) reflected, ‘hopefully the kind of the fuss that we kicked up, has put the school in a position now where they can understand…’.

### Main (contextual) category 5: Social contexts and shaping characteristics

Social transitions were located within other social contexts. Zac (young person, age 18) described how other shared transitions led to a more serious tone in peer conversations, facilitating his gender identity disclosure: ‘I would say that that transition between schools and lockdown was probably the major push’.

Participants also reflected on how personal and social characteristics shaped social transition, including others' reactions. Zac felt that his parents' experiences of prejudice related to their own gay identities had shaped their understanding of his gender identity: ‘…there was definitely some sort of understanding they would have had’, but he also highlighted the uniqueness of the trans experience. Zac also considered how privilege associated with his white ethnicity likely supported his access to services, reflecting: *‘that just, I guess, opened more doors’*; ‘… it puts us more like as a priority…’.

### Interaction of categories (Figure 1)

Following the initial stage of ‘recognising and accepting this young person’, the processes of the other main categories were not processed linearly. A double arrow depicts that ‘negotiating the changes of social transition…’ and ‘negotiating relationships…**’** co‐occurred and were mutually influencing. Arrows from ‘adaptation and resilience’ back to ‘recognising and accepting this young person’ convey that adaptation and resilience developed both as a result of the other aspects of the social transition and supported the social transition to continue and repeat, including in other contexts. As the social transitions occurred across many settings, the overall process typically took place multiple times, beginning with immediate family and then beyond, including within extended family, school, wider communities and public arenas. All aspects of the process were also shaped by contextual factors, represented with the positioning of ‘social contexts and shaping characteristics’ on an outer circle. The circles have a dashed exterior line, representing the permeability of the social transition processes to these contexts and characteristics.

## Discussion

The developed theory explained the social transition of GDYP within the UK family context as a family resilience process. Accounts indicated that it was largely due to difficult experiences, which can be conceptualised as gender minority stressors, that this family resilience was required. Echoing previous evidence, gender minority stressor exposure appeared to negatively impact well‐being, and frequently occurred in schools (Brecht et al., 2024; Hatchel et al., 2019; Meyer, 2003). Schools did, however, appear to have significant potential to be affirming environments.

A salient strand aligning with other research was the centrality of families in the social transitions and the importance of family acceptance in enabling social transition (Bull & D'Arrigo‐Patrick, 2018; Schimmel‐Bristow et al., 2018). Participating families clearly viewed social transition as having a key role in well‐being, describing many examples of associated positive effects. These impacts echo findings from other recent research, including associations between higher perceived transition progress and lower mental health symptoms (Thoma, C, Ej, S., & Salk, 2023).

Notably, there has been some debate regarding the evidence base for well‐being and social transition, with some mixed results (Durwood et al., 2017; Hall et al., 2024; Morandini et al., 2023). However, given the apparent salience of gender minority stress, it seems possible that this may undermine some potential positive impacts of social transition. Exploration of this would be a useful area for future research.

### Implications

Given the salience of gender minority stressors, we recommend these are carefully attended to within assessment and treatment offered to GDYP in mental health and gender services. The need for services sensitive to the needs of these young people seems particularly pressing within the recent UK context, as there appears to have been further increased public and media debate regarding the lives and identities of GDYP since the commencement of the Cass Review (Fox, 2024). A focus on gender minority stress may also open up a more systemic understanding of gender dysphoria as related to non‐accepting social contexts. This would align with other recent research. For example, Brecht et al. (2024) found that experiencing trans hostility was associated with higher rates of gender dysphoria in transgender adolescents.

Building upon the significance of the family in social transitions, a further recommendation is for assessment and treatment to be consistently offered at the family level. This could perhaps include systemic family therapy interventions around themes of changing family needs due to alterations in relational closeness, supporting families to navigate changes and to bolster family functioning.

Considering the apparent potential for schools to affirm and support social transition, as well as the many experiences of non‐affirmation, we recommend school support as another area that needs addressing. Indeed, given the positive experiences of the participants after social transition at school, support for social transition (where desired by GDYP) seems an important area to address for GDYP well‐being. The proposed educational guidance on supporting ‘Gender Questioning Children’, however, raises questions over the extent to which this support for transitioning may be possible in the future; UK educational settings will not have a duty to support social transition, and a ‘cautious approach’ is recommended where requests are accommodated (Department for Education, 2023). We therefore recommend that longer‐term impacts of this new guidance on the ability to socially‐transition and its possible effect on GDYP's well‐being is a continuing focus for research.

Whilst we have focused here on the recent UK context, it seems likely that this will have some international generalisability; indeed, the relevance of UK developments to US clinicians has been highlighted (Knopf, 2024).

### Limitations

This was an all‐white sample, and it is imperative that the voices of people with a range of racial backgrounds are included in theory development. This lack of racial diversity is also particularly limiting given the prominence of gender minority stressors, as minority stressors related to different aspects of identity are considered to intersect (Parent, DeBlaere, & Moradi, 2013; Prendergast & MacPhee, 2018). Additionally, as all the young people had transitioned to a binary gender identity, the extent to which this model applies to GDYP of other gender identities remains unknown. Finally, all findings came from families in which at least one parent was affirming of their GDYPs' identity. The lack of inclusion of less accepting families narrows the generalisability of the presented model significantly; it remains unclear if and how families would engage in a family‐resilience process of social transition without the presence of an affirming parental figure. It would be helpful for future research to build upon these areas by examining the experiences of families representing broader characteristics.

## Conclusion

The current study addressed a previous lack of research, providing further understanding of the experience of GDYP and their families, and contributing a developed model of social transition within the family context. The developed theory explained social transition within the family as a young person‐led family‐resilience process. Continuing development in this area is much needed.

## Conflict of interest statement

The authors have declared that they have no competing or potential conflicts of interest.

## Funding information

No external financial support was received.

## Ethical considerations

Informed consent was obtained for all participants aged 16 and over, and assent was obtained, with parental consent, for participants under 16. HRA/Health and Care Research Wales approval was granted on 10/01/2022 (REC reference: 21/LO/0854). The relevant permissions were obtained for the use and publication of the data presented in Table 1.

## Data Availability

Research data are not shared.
